# Importance of younger age group and high inflammatory status in the association between periodontal disease and diabetes mellitus: results from the Korea National Health and Nutrition Examination Survey 2012–2018

**DOI:** 10.4178/epih.e2024088

**Published:** 2024-11-15

**Authors:** Hyunmin Lee, Myung-Hee Shin

**Affiliations:** Department of Social and Preventive Medicine, Sungkyunkwan University School of Medicine, Suwon, Korea

**Keywords:** Periodontal disease, Diabetes mellitus, Age, Inflammation, C-reactive protein

## Abstract

**OBJECTIVES:**

Although previous studies have demonstrated an association between periodontal disease (PD) and diabetes mellitus (DM), the influence of age and the mediating role of inflammation have seldom been explored. This study investigated this association while considering the modifying effects of age and inflammatory status.

**METHODS:**

This study included 29,491 participants from the 2012–2018 Korea National Health and Nutrition Examination Survey. The community periodontal index (CPI) was assessed by trained dentists using the World Health Organization CPI probe. PD was defined as a CPI score of 3 or 4. Pre-existing and incident DM were identified based on serum glucose levels, a history of DM diagnosis, medication use, and insulin injections. Serum high-sensitivity C-reactive protein (hs-CRP) levels were utilized as an indicator of chronic inflammation.

**RESULTS:**

PD and DM exhibited a significant association, which was more pronounced with incident DM than with pre-existing DM, particularly in individuals younger than 65 years. Among those aged 20–44 years, the odds ratio of incident DM for CPI=4 versus CPI=0 was 2.61 (95% confidence interval, 1.16 to 6.09). High hs-CRP levels (>3 mg/L) were also associated with DM, especially in individuals with PD. This association was stronger with incident DM than with pre-existing DM. A notable joint effect was observed in younger individuals and those with PD.

**CONCLUSIONS:**

The association between PD and DM was more pronounced in younger age groups and those with higher levels of inflammation. Therefore, early interventions for PD in younger patients may be crucial for preventing DM.

## GRAPHICAL ABSTRACT

[Fig f1-epih-46-e2024088]

## Key Message

• The association between PD and DM was more pronounced in younger age groups and those with higher levels of inflammation.

• The odds of DM increased synergistically for those with both PD and high inflammatory status.

• Therefore, early interventions for PD in younger patients may be crucial for preventing DM.

## INTRODUCTION

Diabetes mellitus (DM) and periodontal disease (PD) are highly prevalent chronic diseases in Korea. According to a fact sheet from the Korean Diabetes Association, approximately 1 in 7 adults (13.8%) over 30 years of age have DM. The prevalence of DM is 3.7% among those in their 30s, increasing sharply to 13.0% in the 40s age group. For those aged 65 and older, the prevalence rises to 28.8% [1]. In 2014, the prevalence of PD in Korea was reported at 41.1% [2]. Among individuals in their 20s, PD prevalence was 5.2%, climbing to 16.6% in those in their 30s. The prevalence continues to rise with age, reaching 34.7% in the 40s, 51.1% in the 50s, 66.7% in the 60s, and 84.5% in those aged 70 and above.

PD is a known complication of DM [3]; however, it is less widely known that PD can also increase the risk of DM. A previous systematic review highlighted that while numerous studies have documented an increased risk of DM due to PD, and vice versa, the evidence supporting the former is limited [4]. Additionally, few studies have explored how age modifies this association. Given that the prevalence of PD surges in individuals in their 30s and DM prevalence rises in the 40s, it is crucial to determine whether the relationship between PD and DM is consistent across different age groups.

Several mechanisms have been proposed to explain the relationship between PD and DM, primarily focusing on inflammation and immune responses [5]. PD is caused by periodontal bacteria, which can then spread and induce systemic inflammation [6]. This low-grade systemic inflammation plays a critical role in the development of DM. Conversely, a high inflammatory state, such as that seen in DM, can damage periodontal tissues and exacerbate PD, illustrating the bidirectional nature of the PD-DM relationship [7]. Examining the relationship between PD and DM, either independently or in conjunction with inflammation status, would improve our understanding of the pathway from PD to inflammation to DM. This study aimed to investigate the association between PD and DM, taking into account the modifying effects of age and inflammatory status.

## MATERIALS AND METHODS

### Participants

This study utilized data from the fifth, sixth, and seventh Korea National Health and Nutrition Examination Surveys (KNHANES) conducted between 2012 and 2018, which included information on PD. The KNHANES, a cross-sectional study, was administered by the Ministry of Health and Welfare using a multistage probability cluster sampling design to enhance the representativeness of the sample and the accuracy of the estimates. Consequently, it is possible to generalize the results to the Korean population by employing an analytical method that accounts for the sampling design. Data on basic characteristics and oral health from the KNHANES database (DB) were merged for this analysis. Between 2012 and 2018, a total of 45,493 participants were surveyed. Of these, 9,920 individuals under the age of 20 were excluded. After further excluding those from the remaining 35,573 participants for whom information on DM and PD was unavailable, 29,491 individuals were included in the final analysis.

### Variables

#### Demographic and lifestyle information

Covariates were included in the analysis based on data from the KNHANES DB. This survey encompasses the entire nation and annually involves approximately 10,000 participants. It collects information on a variety of factors including socioeconomic status, health-related behaviors, quality of life, healthcare utilization, anthropometric measurements, biochemical and clinical data for non-communicable diseases, and dietary intake. The survey consists of 3 main components: health interviews, health examinations, and nutrition surveys [8]. Data regarding age, sex, educational level, occupation, alcohol consumption, smoking status, physical activity, and family history of DM were collected through a health survey conducted at a mobile screening center. Obesity status was assessed using body mass index (BMI), which was calculated from body measurements. Data on oral hygiene-related variables were gathered through interviews.

Educational level was classified into four categories: elementary, middle, high, and college graduation. Occupation was divided into three categories: office job, manual labor, and unemployed. Alcohol intake was categorized based on frequency, as either more than or less than once per month. Smoking status was categorized as never smoker, former smoker, or current smoker. Obesity was classified according to the World Health Organization (WHO) standards into three categories: underweight (<18.5 kg/m^2^), normal (18.5 to <25.0 kg/m^2^), and obese (≥25.0 kg/m^2^). For physical activity, the metabolic equivalent value was calculated and categorized based on the WHO-recommended exercise amount, which is more than 150 minutes of moderate-intensity exercise per week. The family history of DM was categorized as either yes or no, considering the history of parents and siblings. Oral hygiene scores were classified on a scale of 0–2 points, with 1 point added for brushing more than twice per day and another for using secondary oral hygiene tools [9]. According to a previous study, there is a significant difference in PD prevalence with respect to having more than 20 teeth [10]. Therefore, the number of remaining teeth was calculated and included in the analysis. Hypertension was defined using systolic blood pressure, diastolic blood pressure and antihypertensive drugs usage. Energy intake was determined using a food intake survey, specifically an individual 24-hour retrospective survey.

#### PD status

PD was defined using periodontal tissue variables obtained from the oral examinations in the KNHANES. These oral examinations included health interviews and assessments. The health examination components covered dental and prosthetic conditions, treatment needs, and prosthetic requirements, adhering to WHO oral examination guidelines. The study was carried out by an oral epidemiological investigator (public health dentist) from the Korea Centers for Disease Control and Prevention (KCDC; currently the Korea Disease Control and Prevention Agency), with additional support from a public health dentist at the city and provincial level.

PD prevalence was determined using a specific PD prevalence variable. The periodontal tissue status for each tooth was evaluated using the WHO CPI probe. The scoring was as follows: “0” indicated healthy periodontal tissue, “1” indicated periodontal tissue with bleeding upon probing, “2” indicated periodontal tissue with calculus or other periodontal anomalies, “3” indicated shallow periodontal pockets with a probing pocket depth (PPD) of 4.0–5.0 mm, and “4” indicated deep periodontal pockets with a PPD of more than 5.0 mm. The highest CPI score obtained was used as the representative value for each participant [11]. Individuals with a CPI score of 3 or 4 were classified as having PD.

#### DM

Blood collection was conducted only after verifying the fasting period by documenting the last intake and the current test time on the survey table. Individuals with a fasting blood sugar level of ≥126 mg/dL, a glycated hemoglobin level of ≥6.5%, a medical diagnosis of DM, or those using blood sugar-lowering drugs or insulin were identified as patients with prevalent DM. This group was further categorized into incident and pre-existing DM. In cases where there was no prior medical diagnosis, but the criteria for fasting blood sugar and glycated hemoglobin levels were met, DM was considered to have developed relatively recently and was thus classified as incident DM. The remaining patients were categorized as having pre-existing DM. Within the pre-existing DM group, if the blood sugar level was <126 mg/dL and the glycated hemoglobin level was <6.5%, the condition was defined as well-controlled DM. Conversely, participants with a blood sugar level ≥126 mg/dL or a glycated hemoglobin level ≥6.5% within the pre-existing DM group were defined as having poorly controlled DM. The duration of DM was calculated by subtracting the age at diagnosis from the current age. Those with a prevalence period of less than 5 years were also defined as incident DM cases.

#### Inflammatory level

C-reactive protein levels were categorized into three groups using HE_hsCRP, the variable derived from the high-sensitivity C-reactive protein (hs-CRP) test (Roche, Mannheim, Germany). The detection range spanned from 0.15 mg/L to 20.00 mg/L. According to the Centers for Disease Control and Prevention/American Heart Association criteria, the hs-CRP levels were classified as: <1, ≥1 but <3, and >3 mg/L.

### Statistical analysis

All analyses were conducted using SAS version 9.4 (SAS Institute Inc., Cary, NC, USA). Weights were calculated using a multistage probability cluster sampling method, and the analyses utilized SurveyLogistic and SurveyFreq. To compare general characteristics, chi-square tests were performed on categorical data, and the Student t-test was used for continuous data. A multivariate logistic regression analysis was conducted to calculate the odds ratios (ORs) and confidence intervals (CIs).

A stratification analysis was conducted by dividing the patients into three age groups: 20–44, 45–64, and 65 years and older. The cutoff age of 45 was established based on a study that determined the appropriate age threshold at which to recommend evaluation for PD [12]. The cutoff age of 65 was chosen because this group is classified as the elderly population in Korea.

### Ethics statement

The 2012–2018 KNHANES was approved by the Institutional Review Board of the KCDC (2012-01EXP-01-2C, 2013-07CON-03-4C, 2013-12EXP-03-5C, 2018-01-03-P-A). The review was waived for the years 2015-2017 under Article 1 (1) of the Bioethics and Safety Act and Article 2 (2)-1 of the Enforcement Decree of the Bioethics and Safety Act.

## RESULTS

### Participants characteristics

Of the 29,491 participants, 25,441 were in the non-diabetic (without DM) group, and 4,050 were in the diabetic (DM) group (Table 1). The average age was 44.94 years in those without diabetes and 59.58 years in the diabetic group. The proportion of men was significantly higher in the DM group compared to those without diabetes (p<0.001). Significant differences were also observed between the two groups in terms of educational level, occupation, alcohol intake, smoking status, and obesity rates (p<0.001). Both the rate of failure to meet the WHO physical activity guidelines and the rate of having a family history of diabetes were higher in the DM group (p<0.001). Additionally, the proportion of patients with low oral hygiene scores was greater in the DM group than in those without diabetes. The percentage of individuals with fewer than 20 remaining teeth was also higher in the DM group (p<0.001). The DM group had a significantly higher proportion of hypertension compared to those without diabetes (p<0.001). Energy intake was lower in the DM group than in those without diabetes (p<0.001).

### Association between periodontal disease and diabetes mellitus

Table 2 illustrates the relationship between PD and DM. A significant association was found between PD and prevalent DM (OR, 1.51; 95% CI, 1.35 to 1.69). Similarly, a significant association was observed between PD prevalence and incident DM (OR, 1.74; 95% CI, 1.47 to 2.07). However, the association between PD and well-controlled pre-existing DM did not reach statistical significance (OR, 1.26; 95% CI, 0.96 to 1.67). In contrast, a significant association was noted for poorly controlled DM (OR, 1.36; 95% CI, 1.17 to 1.58).

The association between CPI 3, 4 and prevalent DM was significant (OR, 1.45; 95% CI, 1.20 to 1.70; OR, 1.93; 95% CI, 1.60 to 2.35; respectively). A significant association was also observed between CPI 1 and prevalent DM (OR, 1.28; 95% CI, 1.01 to 1.61). For incident DM, significant associations were found with CPI 3 and 4 (OR, 1.65; 95% CI, 1.29 to 2.12; OR, 2.43; 95% CI, 1.84 to 3.21; respectively), but not with CPI 1 (OR, 1.02; 95% CI, 0.69 to 1.50). Similar to prevalent DM, associations were observed between CPI 1, 3, 4 and poorly controlled DM. No association was found when considering well-controlled DM.

### Association between community periodontal index and diabetes mellitus by diabetes mellitus disease duration

The results of the stratified analysis by DM disease duration are presented in Table 3. A DM duration of less than 5 years or between 5 years and 14 years was significantly associated with CPI 3 and CPI 4. Conversely, a DM duration of 15 years or more was significantly associated with CPI 1.

### Association between community periodontal index and diabetes mellitus by age

Table 4 presents the results of the analysis stratified by age. There was a significant association between CPI 3, 4 and prevalent DM among those aged 20–44 years and 45–64 years, with the exception of CPI 4 in the 20–44 year age group. For incident DM, a significant association was observed with CPI 3 and 4 in both the 20–44 years and 45–64 years age groups. However, no significant association was found among those aged 65 years and older.

### Association between high-sensitivity C-reactive protein level and diabetes mellitus by periodontal disease status and age

Table 5 illustrates the relationship between hs-CRP levels and DM, stratified by PD status. In cases of prevalent DM, incident DM, and poorly controlled DM, the OR increased with increasing hs-CRP levels, and the ORs were consistently higher in the PD group compared to the non-PD group. The combined effect of PD and elevated hs-CRP levels on DM was greater than the sum of effects of each condition alone. Furthermore, a higher OR was noted for incident DM compared to prevalent DM. No significant association was found in well-controlled DM.

Table 6 illustrates the joint association of hs-CRP levels and PD status with DM, stratified by age. The relationship between PD and DM was most pronounced in individuals aged 20–44, showing a stronger association in this age group compared to others, even without elevated hs-CRP levels (OR for DM in PD, 2.81; 95% CI, 1.46 to 5.43). Similarly, the association between hs-CRP levels and DM was strongest in the 20–44 age group, independent of PD status (OR for DM with hs-CRP>3 mg/L, 2.35; 95% CI, 1.20 to 4.62). These associations were more marked when considering only incident cases of DM. Additionally, when individuals had both PD and hs-CRP levels greater than 3 mg/L, the OR for DM increased synergistically.

## DISCUSSION

In this study, we examined the relationship between PD and DM. We found that the association was stronger with a higher CPI than with a lower one, with incident DM compared to pre-existing DM, in the younger age group (<65) than in the older age group (≥65), and in the higher hs-CRP group (>3 mg/L) than in the lower hs-CRP group. Prevalent DM was significantly associated with a CPI of 1, and this association became stronger as PD severity increased to CPI levels 3 and 4. Incident DM demonstrated stronger associations with CPI levels 3 and 4 than did pre-existing DM, indicating that advanced PD might elevate the risk of DM.

The association between PD and incident DM was found to be stronger in younger age groups (aged 20–44 and 45–64) compared to the older age group (aged ≥65). Adding hs-CRP levels to the analysis of the PD and incident DM association revealed that PD significantly increased the odds of incident DM in individuals aged 20–44, even without elevated hs-CRP levels (OR, 6.58; Table 6). For those with both PD and high hs-CRP levels (>3 mg/L), the odds of incident DM increased synergistically (OR, 23.31). This suggests that PD might independently be associated with incident DM and could serve as a strong marker of systemic inflammation, particularly in conjunction with hs-CRP, especially among younger individuals who typically have a stronger immune response. Tables 5 and 6 show larger differences in the proportions of high hs-CRP levels (>3 mg/L) between the DM group and the non-DM group among those with incident DM, poorly controlled pre-existing DM, PD, and younger age. These findings suggest that controlling PD from a younger age may be important in preventing DM.

In patients with PD, levels of inflammatory mediators in the plasma are also elevated [13,14]. Systemic inflammation associated with local inflammation caused by periodontal infections can lead to insulin resistance [15]. Typically, patients with periodontitis exhibit increased levels of the inflammatory mediator tumor necrosis factor (TNF)-alpha, which interferes with insulin signal transmission, thereby promoting insulin resistance [16]. This mechanism is corroborated by studies showing that treatment of PD, which reduces inflammatory mediators, improves glycated hemoglobin levels [17]. At a younger age, this mechanism may be more pronounced due to increased metabolism; thus, the risk of DM due to inflammation may be higher in younger individuals. Demmer & Collegues [18,19] also found a slightly stronger association between incident DM and higher periodontal indices in individuals under 50 years of age compared to those 50 years and older, in their study using the United States National Health and Nutrition Survey cohort.

In patients with DM, systemic inflammatory responses are elevated, leading to increased blood levels of inflammatory mediators such as Interleukin (IL)-6, TNF-alpha, and IL-1 [20]. Additionally, oxidative stress and apoptosis are also heightened [20,21]. These reactions can cause damage to periodontal tissues [22]. In this study, CPI 1 was exclusively associated with poorly controlled DM and prevalent DM with a disease duration of 15 years or more, suggesting that longstanding DM may increase the risk of PD. Moreover, the relationship between the two diseases was not significant in well-controlled DM but was significant in poorly controlled DM, aligning with findings from previous studies [23,24]. Consequently, our findings indicate a potential bidirectional relationship between PD and DM.

This study has several limitations. Firstly, due to its cross-sectional nature, establishing a causal relationship was not possible. Nevertheless, by analyzing incident DM and categorizing patients based on the duration of DM, we aimed to assess the directionality of the association. The findings align with those from previous cohort studies. Secondly, we were unable to differentiate between types of DM, as the KNHANES DB does not distinguish between type 1 and type 2 DM. It is important to note, however, that type 1 DM accounts for less than 2% of all DM cases in Korea [25]. Thirdly, hs-CRP level data were not available for all participants. Despite this, the representativeness of the hs-CRP analysis was maintained through the use of a multistage probability cluster sampling design for weighting.

This study boasts several strengths. Firstly, it utilizes data from the KNHANES, which is highly representative of the Korean population due to its large sample size and sampling design. The analysis incorporates elements of the multistage probability cluster sampling design, allowing the results to be generalized to the Korean population. Secondly, the assessment of all PD statuses was conducted by licensed dentists, thereby enhancing the validity of the data. Thirdly, the analyses accounted for various stages of DM and PD, facilitating the inference of potential temporal sequences. Fourthly, the adjustment for various potential confounding factors was achieved using variables from the KNHANES DB. Finally, the use of hs-CRP levels in the analysis may have improved the understanding of how inflammation mediates the relationship between PD and DM, particularly in younger age groups.

In conclusion, the association between PD and DM is more pronounced in younger age groups and those with higher levels of inflammation. Therefore, initiating early intervention for PD from a younger age may be crucial for preventing DM.

## Figures and Tables

**Figure f1-epih-46-e2024088:**
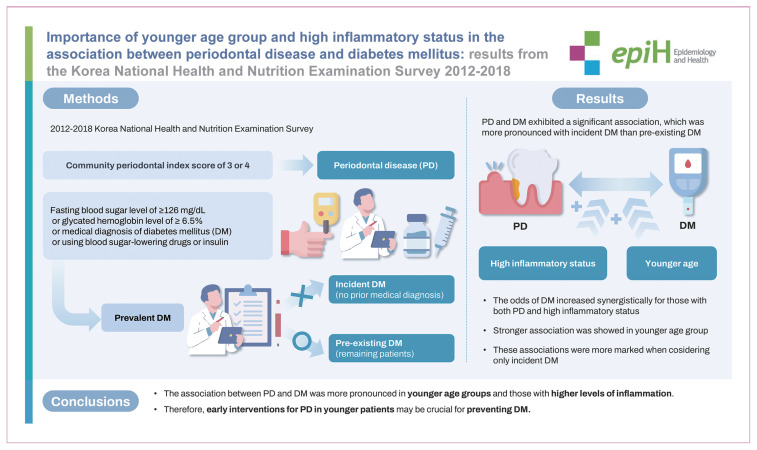


**Table 1:** Characteristics of participants from 2012 to 2018 Korea National Health and Nutrition Examination Survey by DM status

Characteristics	DM		p-value^1^
	No	Yes	
Total	25,441 (88.4)	4,050 (11.6)	
Average age (yr)	44.94±0.17	59.58±0.27	<0.001
Sex			<0.001
Male	10,675 (47.6)	2,047 (54.2)	
Female	14,766 (52.4)	2,003 (45.8)	
Education level			<0.001
≤Elementary school graduation	4,424 (13.5)	1,507 (35.7)	
Middle school graduation	2,348 (8.5)	619 (15.9)	
High school graduation	8,576 (38.5)	1,029 (28.6)	
≥College graduation	9,588 (39.5)	685 (19.7)	
Occupation			<0.001
Office job	6,576 (28.0)	481 (14.5)	
Manual labor	9,125 (37.3)	1,456 (39.5)	
Unemployed	9,199 (34.7)	1,904 (46.0)	
Alcohol intake (times/mo)			<0.001
<1	11,303 (40.8)	2,114 (50.1)	
≥1	13,973 (59.2)	1,810 (49.9)	
Smoking status			<0.001
Never	15,665 (58.1)	2,114 (51.2)	
Former	4,929 (19.6)	1,041 (26.0)	
Current	4,674 (22.3)	756 (22.8)	
Obesity (BMI, kg/m^2^)			<0.001
Underweight (<18.5)	1,073 (4.7)	44 (1.1)	
Normal (18.5–25.0)	16,372 (64.5)	1,959 (47.9)	
Obese (≥25.0)	7,872 (30.9)	2,039 (50.9)	
Physical activity (WHO guideline)		<0.001	
No	18,064 (67.7)	3,075 (77.0)	
Yes	6,858 (32.3)	762 (23.0)	
Family history of DM			<0.001
No	18,458 (79.0)	2,055 (59.0)	
Yes	5,068 (21.0)	1,418 (41.0)	
Oral hygiene score			<0.001
0	1,742 (5.7)	599 (15.6)	
1	10,838 (43.4)	2,016 (48.7)	
2	12,861 (50.1)	1,435 (35.7)	
No. of remaining teeth			<0.001
<20	2,602 (7.6)	1,083 (24.3)	
≥20	22,839 (92.4)	2,967 (75.7)	
Hypertension			<0.001
No	12,621 (53.2)	725 (21.0)	
Prehypertension^2^	6,220 (24.6)	796 (22.6)	
Hypertension^3^	6,531 (22.1)	2,403 (56.4)	
Energy intake (kcal)	2,078.24±9.60	1,880.20±20.07	<0.001

Values are presented as number (%) or mean±standard error.

DM, diabetes mellitus; BMI, body mass index; WHO, World Health Organization.

1 Calculated from the chi-square test for categorical variables and from generalized linear regression analysis for continuous variables.

2 Non-hypertensive people with systolic blood pressure 120–139 mmHg or diastolic blood pressure 80–89 mmHg.

3 People with systolic blood pressure ≥140 mmHg or diastolic blood pressure ≥90 mmHg or antihypertensive drugs usage.

**Table 2:** Association between PD status identified from the CPI value and status of DM

Diseases status	Prevalent DM				Incident DM^1^			Pre-existing DM well controlled^2^			Pre-existing DM poorly controlled^3^		
	No	Yes	Crude	Adjusted^4^	Yes	Crude	Adjusted^4^	Yes	Crude	Adjusted^4^	Yes	Crude	Adjusted^4^
PD													
No	18,359 (74.6)	2,042 (50.1)	1.00 (reference)	1.00 (reference)	767 (50.3)	1.00 (reference)	1.00 (reference)	271 (52.2)	1.00 (reference)	1.00 (reference)	1,004 (49.5)	1.00 (reference)	1.00 (reference)
Yes	7,082 (25.4)	2,008 (49.9)	2.92 (2.67, 3.18)^*^	1.51 (1.35, 1.69)^*^	710 (49.7)	2.90 (2.54, 3.31)^*^	1.74 (1.47, 2.07)^*^	248 (47.8)	2.69 (2.16, 3.35)^*^	1.26 (0.96, 1.67)	1,050 (50.5)	2.99 (2.66, 3.37)^*^	1.36 (1.17, 1.58)^*^
CPI													
0	7,755 (31.2)	806 (19.4)	1.00 (reference)	1.00 (reference)	285 (18.2)	1.00 (reference)	1.00 (reference)	120 (21.9)	1.00 (reference)	1.00 (reference)	401 (19.9)	1.00 (reference)	1.00 (reference)
1	1,685 (6.3)	272 (6.1)	1.55 (1.29, 1.87)^*^	1.28 (1.01, 1.61)^*^	77 (4.5)	1.23 (0.88, 1.73)	1.02 (0.69, 1.50)	42 (7.3)	1.65 (1.09, 2.50)	1.05 (0.60, 1.84)	153 (7.1)	1.78 (1.40, 2.25)^*^	1.51 (1.12, 2.05)^*^
2	8,919 (37.1)	964 (24.6)	1.07 (0.94, 1.21)	1.05 (0.89, 1.23)	405 (27.5)	1.27 (1.04, 1.56)^*^	1.14 (0.89, 1.45)	109 (23.0)	0.88 (0.64, 1.22)	0.90 (0.63, 1.30)	450 (22.5)	0.95 (0.80, 1.13)	0.99 (0.79, 1.23)
3	5,274 (18.9)	1,330 (32.8)	2.79 (2.47, 3.15)^*^	1.45 (1.20, 1.70)^*^	474 (32.2)	2.93 (2.41, 3.55)^*^	1.65 (1.29, 2.12)^*^	166 (32.8)	2.48 (1.87, 3.27)^*^	1.10 (0.77, 1.55)	690 (33.2)	2.76 (2.33, 3.27)^*^	1.34 (1.09, 1.65)^*^
4	1,808 (6.6)	678 (17.1)	4.18 (3.61, 4.86)^*^	1.93 (1.60, 2.35)^*^	236 (17.5)	4.56 (3.67, 5.66)^*^	2.43 (1.84, 3.21)^*^	82 (15.0)	3.24 (2.28, 4.61)^*^	1.48 (0.96, 2.28)	360 (17.3)	4.14 (3.39, 5.05)^*^	1.63 (1.26, 2.10)^*^

Values are presented as number (%) or odds ratio (95% confidence interval).

PD, periodontal disease; CPI, community periodontal index; DM, diabetes mellitus; HbA1c, glycosylated hemoglobin A1C; KNHANES, Korea National Health and Nutrition Examination Survey.

1 People with no history of DM with current fasting blood glucose level ≥126 mg/dL or HbA1c ≥6.5% in their KNHANES examination.

2 People with previous history of DM with current fasting blood glucose level <126 md/dL and HbA1c <6.5% in their KNHANES examination.

3 People with previous history of DM with current fasting blood glucose level≥ 126 mg/dL or HbA1c ≥6.5% in their KNHANES examination.

4 Adjusted for age, sex, educational level, occupation, alcohol intake, smoking status, obesity, physical activity, energy intake, family history of DM, oral hygiene score, hypertension and number of remaining teeth.

* p<0.05.

**Table 3:** Association between periodontal disease status identified from the highest CPI value and prevalent DM by DM disease duration

DM disease duration (yr)^1^	CPI	Prevalent DM		Crude	Adjusted^2^
		No	Yes		
<5	0	7,755 (31.2)	476 (18.2)	1.00 (reference)	1.00 (reference)
	1	1,685 (6.3)	162 (5.7)	1.56 (1.22, 1.98)^*^	1.29 (0.96, 1.74)
	2	8,919 (37.1)	643 (26.5)	1.23 (1.04, 1.44)^*^	1.13 (0.93, 1.39)
	3	5,274 (18.9)	796 (32.0)	2.91 (2.50, 3.39)^*^	1.53 (1.25, 1.86)^*^
	4	1,808 (6.6)	409 (17.6)	4.59 (3.84, 5.48)^*^	2.20 (1.76, 2.76)^*^
5–14	0	7,755 (31.2)	211 (21.3)	1.00 (reference)	1.00 (reference)
	1	1,685 (6.3)	58 (5.5)	1.28 (0.90, 1.83)	0.97 (0.64, 1.48)
	2	8,919 (37.1)	216 (23.0)	0.91 (0.71, 1.15)	0.96 (0.71, 1.28)
	3	5,274 (18.9)	350 (35.2)	2.73 (2.20, 3.38)^*^	1.37 (1.06, 1.77)^*^
	4	1,808 (6.6)	165 (15.0)	3.35 (2.59, 4.32)^*^	1.48 (1.07, 2.03)^*^
≥15	0	7,755 (31.2)	119 (22.2)	1.00 (reference)	1.00 (reference)
	1	1,685 (6.3)	52 (9.2)	2.05 (1.40, 3.02)^*^	1.69 (1.03, 2.79)^*^
	2	8,919 (37.1)	105 (17.9)	0.68 (0.48, 0.95)^*^	0.72 (0.48, 1.09)
	3	5,274 (18.9)	184 (32.0)	2.38 (1.76, 3.21)^*^	1.15 (0.80, 1.65)
	4	1,808 (6.6)	104 (18.8)	4.01 (2.88, 5.59)^*^	1.51 (0.97, 2.35)

Values are presented as number (%) or odds ratio (95% confidence interval).

CPI, community periodontal index; DM, diabetes mellitus.

1 The DM disease duration was calculated as the difference between the current age and the age at diagnosis.

2 Adjusted for age, sex, educational level, occupation, alcohol intake, smoking status, obesity, physical activity, energy intake, family history of DM, oral hygiene score, hypertension and number of remaining teeth.

* p<0.05.

**Table 4:** Association between periodontal disease status identified from the highest CPI value and DM status by age

Age (yr)	CPI	Prevalent DM		Adjusted^1^	Incident DM^2^	Adjusted^1^
		No	Yes		Yes	
20–44	0	4,193 (37.5)	91 (23.5)	1.00 (reference)	59 (21.4)	1.00 (reference)
	1	803 (7.1)	18 (5.2)	0.96 (0.48, 1.93)	11 (4.4)	0.75 (0.28, 2.01)
	2	4,609 (43.1)	138 (37.9)	1.07 (0.73, 1.56)	100 (38.7)	1.20 (0.74, 1.94)
	3	1,122 (10.0)	102 (26.0)	2.32 (1.48, 3.63)^*^	70 (27.5)	2.58 (1.45, 4.59)^*^
	4	251 (2.2)	32 (7.4)	1.86 (0.91, 3.81)	19 (8.0)	2.61 (1.16, 6.09)^*^
45–64	0	2,438 (24.3)	309 (16.4)	1.00 (reference)	118 (15.3)	1.00 (reference)
	1	514 (4.7)	103 (5.0)	1.57 (1.12, 2.19)^*^	32 (3.6)	1.17 (0.66, 2.08)
	2	3,231 (33.6)	440 (24.8)	1.10 (0.87, 1.38)	182 (25.2)	1.23 (0.80, 1.59)
	3	2,584 (26.5)	580 (32.7)	1.51 (1.21, 1.87)^*^	233 (32.5)	1.59 (1.14, 2.20)^*^
	4	989 (10.9)	354 (21.0)	2.33 (1.80, 3.15)^*^	151 (23.5)	2.87 (2.04, 4.02)^*^
≥65	0	1,124 (25.1)	406 (22.0)	1.00 (reference)	108 (21.4)	1.00 (reference)
	1	368 (7.6)	151 (7.8)	1.13 (0.80, 1.59)	34 (6.5)	1.03 (0.57, 1.86)
	2	1,079 (22.0)	386 (19.8)	0.91 (0.71, 1.16)	123 (22.7)	1.07 (0.71, 1.61)
	3	1,568 (33.1)	648 (35.2)	1.12 (0.90, 1.40)	171 (35.9)	1.21 (0.84, 1.74)
	4	568 (12.3)	292 (15.2)	1.17 (0.90, 1.54)	66 (13.5)	1.08 (0.67, 1.74)

Values are presented as number (%) or odds ratio (95% confidence interval).

CPI, community periodontal index; DM, diabetes mellitus; HbA1c, glycosylated hemoglobin A1C; KNHANES, Korea National Health and Nutrition Examination Survey.

1 Adjusted for sex, educational level, occupation, alcohol intake, smoking status, obesity, physical activity, energy intake, family history of DM, oral hygiene score, hypertension and number of remaining teeth.

2 People with no history of DM with current fasting blood glucose level ≥126 mg/dL or HbA1c ≥6.5% in their KNHANES examination.

* p<0.05.

**Table 5:** Association between serum hs-CRP level and status of DM by PD status identified from the highest CPI value, and differences in hs-CRP level by status of DM

PD	hs-CRP	Prevalent DM		Crude	Adjusted^1^	Incident DM^2^	Crude	Adjusted^1^	Pre-existing DM—well controlled^3^	Crude	Adjusted^1^	Pre-existing DM—poorly controlled^4^	Crude	Adjusted^1^
		No	Yes											
No	<1	7,084 (74.9)	660 (62.9)	1.00 (reference)	1.00 (reference)	183 (50.8)	1.00 (reference)	1.00 (reference)	111 (76.0)	1.00 (reference)	1.00 (reference)	366 (67.3)	1.00 (reference)	1.00 (reference)
	1–3	1,699 (18.0)	255 (24.3)	1.65 (1.38, 1.99)^*^	1.26 (0.99, 1.59)	115 (31.9)	2.49 (1.90, 3.26)^*^	1.92 (1.39, 2.66)^*^	22 (15.1)	1.01 (0.61, 1.67)	0.56 (0.28, 1.12)	118 (21.7)	1.41 (1.08, 1.84)^*^	1.07 (0.76, 1.51)
	>3	673 (7.1)	135 (12.9)	2.30 (1.77, 3.00)^*^	1.46 (1.04, 2.06)^*^	62 (17.2)	4.05 (2.78, 5.89)^*^	2.33 (1.50, 3.63)^*^	13 (8.9)	1.13 (0.49, 2.63)	0.65 (0.21, 2.09)	60 (11.0)	1.75 (1.18, 2.60)^*^	1.18 (0.70, 1.44)
	p-value^5^	<0.001				<0.001			0.512			<0.001		
Yes	<1	2,740 (68.6)	639 (57.8)	2.54 (2.21, 2.92)^*^	1.26 (1.06, 1.50)^*^	160 (46.5)	2.33 (1.81, 3.00)^*^	1.56 (1.13, 2.15)^*^	112 (70.0)	2.88 (2.10, 3.94)^*^	0.99 (0.68, 1.43)	367 (61.0)	2.56 (2.13, 3.07)^*^	1.14 (0.90, 1.44)
	1–3	933 (23.3)	313 (28.3)	4.28 (3.48, 5.25)^*^	1.72 (1.31, 2.25)^*^	117 (34.0)	6.70 (4.90, 9.17)^*^	2.86 (1.87, 4.37)^*^	34 (21.3)	2.61 (1.68, 4.04)^*^	0.99 (0.68, 1.43)	162 (26.9)	3.51 (2.72, 4.54)^*^	1.36 (0.98, 1.88)
	>3	324 (8.1)	154 (13.9)	5.86 (4.47, 7.68)^*^	2.87 (1.99, 4.14)^*^	67 (19.5)	9.47 (6.53, 13.73)^*^	5.48 (3.36, 8.93)^*^	14 (8.8)	2.97 (1.62, 5.46)^*^	1.17 (0.52, 2.65)	73 (12.1)	4.83 (3.32, 7.02)^*^	2.11 (1.30, 3.44)^*^
	p-value^5^	<0.001				<0.001			0.813			<0.001		
p for trend					<0.001			<0.001			0.936			0.009

Values are presented as number (%) or odds ratio (95% confidence interval).

hs-CRP, high-sensitivity C-reactive protein; DM, diabetes mellitus; PD, periodontal disease; CPI, comm unity periodontal index; HbA1c, glycosylated hemoglobin A1C; KNHANES, Korea National Health and Nutrition Examination Survey.

1 Adjusted for age, sex, educational level, occupation, alcohol intake, smoking status, obesity, physical activity, energy intake, family history of DM, oral hygiene score, hypertension and number of remaining teeth.

2 People with no history of DM with current fasting blood glucose level ≥126 mg/dL or HbA1c ≥6.5% in their KNHANES examination.

3 People with previous history of DM with current fasting blood glucose level <126 md/dL and HbA1c <6.5% in their KNHANES examination.

4 People with previous history of DM with current fasting blood glucose level ≥126 mg/dL or HbA1c ≥6.5% in their KNHANES examination.

5 Calculated from the chi-square test.

* p<0.05.

**Table 6:** Association between hs-CRP level and DM by PD status identified from highest CPI value and age and differences in hs-CRP level by DM status

Age (yr)	PD	hs-CRP (mg/L)	Prevalent DM		Crude	Adjusted^2^	Incident DM^1^	Crude	Adjusted^2^
			No	Yes			Yes		
20–44	No	<1	3,700 (76.5)	57 (46.0)	1.00 (reference)	1.00 (reference)	28 (34.6)	1.00 (reference)	1.00 (reference)
		1–3	811 (16.8)	40 (32.3)	2.98 (1.84, 4.83)^*^	1.85 (1.06, 3.23)^*^	31 (38.3)	5.52 (2.80, 10.89)^*^	4.47 (2.12, 9.42)^*^
		>3	324 (6.7)	27 (21.8)	5.57 (2.99, 10.34)^*^	2.35 (1.20, 4.62)^*^	22 (27.2)	11.46 (5.25, 25.01)^*^	6.46 (3.03, 13.78)^*^
		p-value^3^	<0.001				<0.001		
	Yes	<1	507 (69.2)	32 (38.1)	4.30 (2.59, 7.15)^*^	2.81 (1.46, 5.43)^*^	19 (33.9)	7.52 (3.62, 15.61)^*^	6.58 (2.83, 13.77)^*^
		1–3	173 (23.6)	28 (33.3)	11.26 (5.93, 21.41)^*^	5.76 (2.69, 12.33)^*^	20 (35.7)	23.05 (9.97, 53.28)^*^	14.33 (5.21, 39.47)^*^
		>3	53 (7.2)	24 (28.6)	22.46 (11.48, 43.96)^*^	7.91 (3.07, 20.34)^*^	17 (30.4)	42.40 (18.58, 96.76)^*^	23.31 (8.39, 64.78)^*^
		p-value^3^	<0.001				<0.001		
	p for trend					<0.001			<0.001
45–64	No	<1	2,477 (76.2)	273 (65.5)	1.00 (reference)	1.00 (reference)	78 (51.7)	1.00 (reference)	1.00 (reference)
		1–3	556 (17.1)	101 (24.2)	1.73 (1.31, 2.29)^*^	1.43 (1.02, 2.01)^*^	49 (32.5)	2.28 (1.47, 3.53)^*^	1.92 (1.18, 3.14)^*^
		>3	217 (6.7)	43 (10.3)	1.98 (1.25, 3.14)^*^	1.62 (0.96, 2.75)	24 (15.9)	3.40 (1.98, 5.84)^*^	2.71 (1.38, 5.34)^*^
		p-value^3^	<0.001				<0.001		
	Yes	<1	1,430 (70.2)	281 (57.4)	1.73 (1.40, 2.14)^*^	1.31 (1.01, 1.69)^*^	89 (48.1)	1.67 (1.16, 2.39)^*^	1.43 (0.96, 2.11)^*^
		1–3	454 (22.3)	149 (30.4)	3.58 (2.66, 4.81)^*^	2.07 (1.44, 2.98)^*^	67 (36.2)	5.96 (3.81, 9.32)^*^	2.87 (1.67, 4.96)^*^
		>3	152 (7.5)	60 (12.2)	4.46 (2.91, 6.84)^*^	3.44 (2.06, 5.74)^*^	29 (15.7)	7.27 (3.99, 13.23)^*^	5.00 (2.35, 10.63)^*^
		p-value^3^	<0.001				<0.001		
	p for trend					<0.001			<0.001
≥65	No	<1	907 (66.2)	330 (64.8)	1.00 (reference)	1.00 (reference)	77 (60.2)	1.00 (reference)	1.00 (reference)
		1–3	332 (24.2)	114 (22.4)	0.95 (0.72, 1.26)	0.78 (0.56, 1.10)	35 (27.3)	1.19 (0.73, 1.94)	0.89 (0.46, 1.71)
		>3	132 (9.6)	65 (12.8)	1.20 (0.82, 1.76)	0.79 (0.46, 1.35)	16 (12.5)	1.40 (0.74, 2.67)	0.56 (0.20, 1.56)
		p-value^3^	<0.001				<0.001		
	Yes	<1	803 (65.4)	326 (61.3)	1.01 (0.82, 1.25)	0.87 (0.66, 1.13)	52 (50.5)	0.68 (0.45, 1.05)	0.69 (0.40, 1.19)
		1–3	306 (24.9)	136 (25.6)	1.16 (0.88, 1.52)	0.87 (0.60, 1.25)	30 (29.1)	1.18 (0.68, 2.04)	0.89 (0.42, 1.91)
		>3	119 (9.7)	70 (13.2)	1.52 (1.04, 2.21)^*^	1.26 (0.76, 2.07)	21 (20.4)	1.87 (1.00, 3.49)	2.07 (0.96, 4.48)
		p-value^3^	<0.001				<0.001		
	p for trend					0.760			0.983

Values are presented as number (%) or odds ratio (95% confidence interval).

hs-CRP, high-sensitivity C-reactive protein; DM, diabetes mellitus; PD, periodontal disease; CPI, community periodontal index; HbA1c, glycosylated hemoglobin A1c; KNHANES, Korea National Health and Nutrition Examination Survey.

1 People with no history of DM with current fasting blood glucose level ≥126 mg/dL or HbA1c ≥6.5% in their KNHANES examination.

2 Adjusted for sex, educational level, occupation, alcohol intake, smoking status, obesity, physical activity, energy intake, family history of DM, oral hygiene score, hypertension and number of remaining teeth.

3 Calculated from the chi-square test.

* p<0.05.
